# An “Uncharacterized” Australian Virus Is the Earliest Known Example of Ross River Virus with Changes in the nsP3 Protein Associated with the Explosive Outbreak of Ross River Virus Infection in the Pacific Region from 1979 to 1980

**DOI:** 10.1128/MRA.00838-21

**Published:** 2021-11-18

**Authors:** John G. Aaskov, Melissa Graham, Wenjun Liu

**Affiliations:** a Australian Defence Force Malaria and Infectious Disease Institute, Enoggera, Australia; b Queensland University of Technology, Brisbane, Australia; c Queensland Institute of Medical Research-Berghofer Medical Research Institute, Brisbane, Australia; KU Leuven

## Abstract

Ross River virus recovered from a South Australian patient during an outbreak of epidemic polyarthritis in 1971 is the earliest known genome sequence with the duplicated 12-amino-acid motif in the nsP3 protein that was found in strains responsible for the outbreak of epidemic polyarthritis in the Pacific region from 1979 to 1980.

## ANNOUNCEMENT

When Ralph Doherty retired as director of the Queensland Institute of Medical Research in 1978, he left a number of “uncharacterized” viruses, one of which was labeled Mannum V. The virus was isolated by injecting material that had been collected during an outbreak of epidemic polyarthritis near Mannum (34°53′59S, 139°17′60E) in South Australia in 1971 (1, 2) intracerebrally into 1-day-old mice. Lack of any reference to mosquitoes suggested that Mannum V was derived from acute-phase serum from a patient.

Suckling mouse brain from an ampoule labeled “Mannum V. 24/6/71” was added to a culture of Aedes albopictus C6-36 (3) cells at 30°C for 7 days before cells and tissue culture supernatant were collected for analysis. Monoclonal antibodies 2A-2C3 (anti-alphavirus) (4) and D7 (anti-Ross River virus [RRV]) (5) but not the anti-flavivirus 4G2 (6) and 6B6C1 (7) antibodies reacted with infected C6-36 cells in indirect immunofluorescence assays, suggesting that the virus was RRV (genus *Alphavirus*, family *Togaviridae*). RNA extracted from culture supernatant using a QIAamp viral RNA minikit (Qiagen) was used for library preparation with a Nextera XT kit at the Australian Genome Research Facility. Sequencing was performed on an Illumina MiniSeq system with 300 cycles in mid-output mode. A total of 17,150,987 sequences, with an average length of 150 nucleotides (nt), were generated. Read quality was assessed using FASTQC. Adapters were trimmed and poor-quality bases were removed using Trim Galore. The sequence data were assembled by aligning the reads to a reference genome, i.e., RRV strain QML1 (GenBank accession number GQ433354), to generate the consensus sequence and to determine the open reading frames (ORFs) and annotations using Geneious R11 v.11.1.2 with default parameters. The average sequence coverage was 82,104 reads/nucleotide. The sequences of the 5′ and 3′ untranslated regions (UTRs) were confirmed by Sanger sequencing of cDNA generated using a 5′/3′ rapid amplification of cDNA ends (RACE) kit (Roche, Germany) as described previously (8).

**FIG 1:**
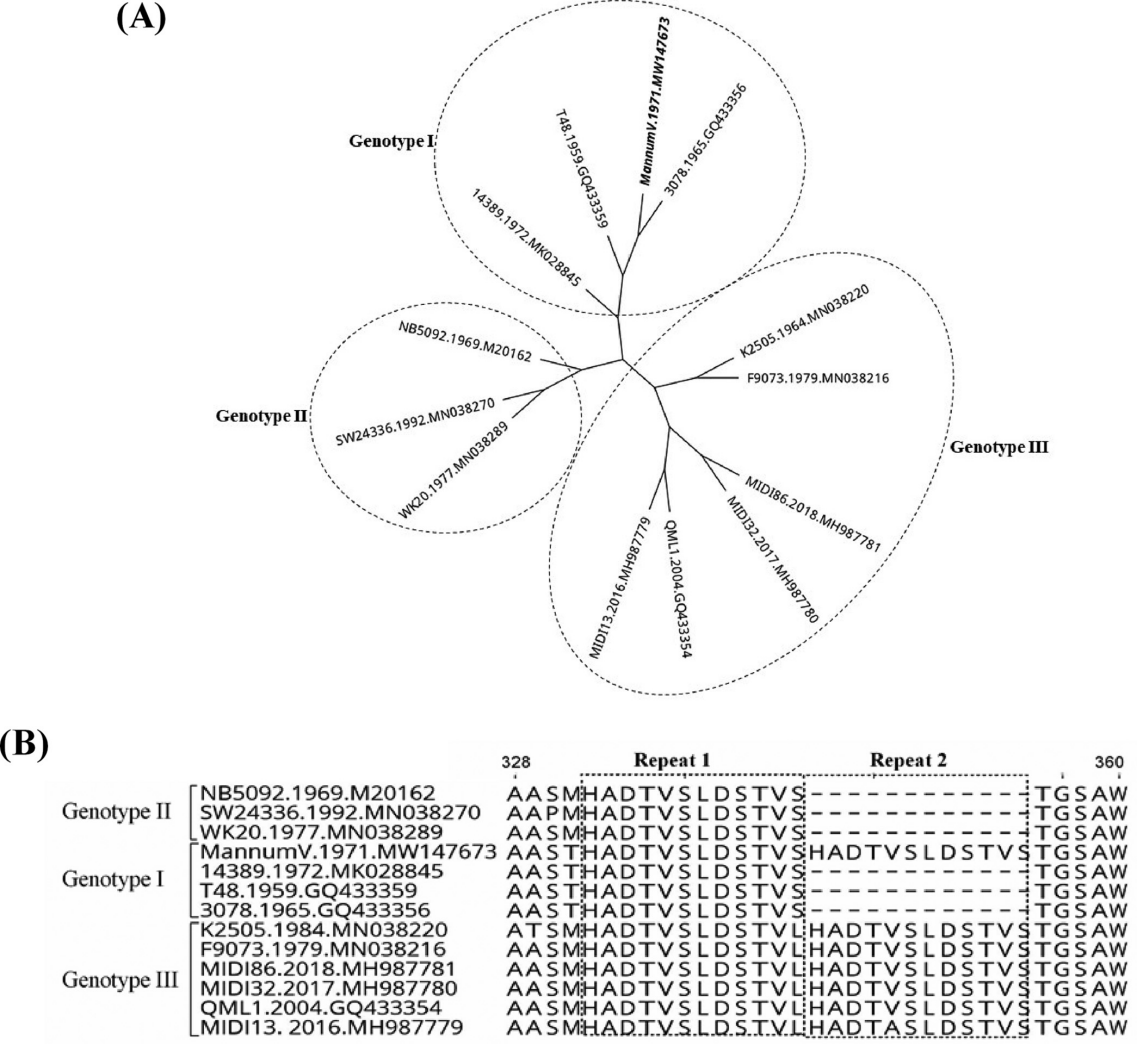
(A) Phylogeny of RRV using whole ORF genomes, classifying the RRV Mannum V strain (highlighted) into genotype I. For each genome, the strain name, year of isolation, and GenBank accession number are indicated. Bayesian phylogenetic analyses were performed with software from Geneious v.11.2, applying the HKY plus gamma substitution model with a gamma molecular clock model of uniform branch lengths, a chain length of 1 million, and a 10% burn-in length. (B) Alignment of amino acids 328 to 360 of the nsP3 proteins of the RRV strains in panel A using Muscle alignment software in Geneious v.11.2.

The genome contained 11,890 nt, excluding the poly(A) tail, and had a G+C content of 51.3%. Phylogenetic comparisons of Mannum V with other selected RRV genomes (8–10) placed it with strains of RRV genotype I recovered from mosquitoes in 1959 (strain T48, GenBank accession no. GQ433359) and birds (strain 3078, GenBank accession no. GQ433356) in 1965 (2) and an isolate recovered from a febrile child in 1972 (strain 14389, GenBank accession no. MK028845) (Fig. 1A) (11). Mannum V is the only known example of a genotype I or II strain of RRV with the amino acid sequence HADTVSLDSTVS duplicated in the hypervariable region of the nsP3 protein (Fig. 1B), whereas this duplication is found in all current genotype III RRVs, which replaced genotypes I and II. The detection of this duplicated motif in a genotype I virus may provide new insights into the evolution of RRV. The largest outbreak of RRV infection in the Pacific region from 1979 to 1980 (12, 13) was due to genotype III viruses. The function of this duplicated element is not clear. It may provide a fitness gain to RRV, possibly through an epistasis interaction with another part of the RRV genome (14, 15).

### Data availability.

Raw next-generation sequencing (NGS) reads were deposited in the Sequence Read Archive (SRA) under accession number PRJNA661132. The RRV Mannum V genome sequence is available in GenBank (accession number MW147673).
